# Development and Genetic Characterization of A Novel Herbicide (Imazethapyr) Tolerant Mutant in Rice (*Oryza sativa* L.)

**DOI:** 10.1186/s12284-017-0151-8

**Published:** 2017-04-04

**Authors:** D. Shoba, M. Raveendran, S. Manonmani, S. Utharasu, D. Dhivyapriya, G. Subhasini, S. Ramchandar, R. Valarmathi, Nitasha Grover, S. Gopala Krishnan, A. K. Singh, Pawan Jayaswal, Prashant Kale, M. K. Ramkumar, S. V. Amitha Mithra, T. Mohapatra, Kuldeep Singh, N. K. Singh, N. Sarla, M. S. Sheshshayee, M. K. Kar, S. Robin, R. P. Sharma

**Affiliations:** 1 Tamil Nadu Agricultural University, Coimbatore, 641003 India; 2 Division of Genetics, ICAR-Indian Agricultural Research Institute, New Delhi, 110012 India; 3 ICAR-National Research Centre on Plant Biotechnology, Pusa, New Delhi, 110012 India; 4 Indian Council of Agriculture Research, New Delhi, 110 001 India; 5 Punjab Agricultural University, Ludhiana, 141004 India; 6 Indian Institute of Rice Research, Rajendranagar, Hyderabad, 500030 India; 7 University of Agricultural Sciences, Bengaluru, 560065 India; 8 National Rice Research Institute, Cuttack, Odisha 753006 India; 9 INSA Honorary Scientist, NRCPB, IARI, Pusa, New Delhi, 110012 India; 10 Present address: ICAR-National Bureau of Plant Genetic Resources, Pusa, New Delhi, 110012 India

**Keywords:** Rice, EMS Mutagenesis, Herbicide tolerance, Imazethapyr, AHAS

## Abstract

**Background:**

Increased water and labour scarcity in major rice growing areas warrants a shift towards direct seeded rice cultivation under which management of weeds is a major issue. Use of broad spectrum non-selective herbicides is an efficient means to manage weeds. Availability of rice genotypes with complete tolerance against broad-spectrum non-selective herbicides is a pre-requisite for advocating use of such herbicides. In the present study, we developed an EMS induced rice mutant, ‘HTM-N22‘, exhibiting tolerance to a broad spectrum herbicide, ‘Imazethapyr‘, and identified the mutations imparting tolerance to the herbicide.

**Results:**

We identified a stable and true breeding rice mutant, HTM-N22 (HTM), tolerant to herbicide, Imazethapyr, from an EMS-mutagenized population of approximately 100,000 M_2_ plants of an upland rice variety, Nagina 22 (N22). Analysis of inheritance of herbicide tolerance in a cross between Pusa 1656-10-61/HTM showed that this trait is governed by a single dominant gene. To identify the causal gene for Imazethapyr tolerance, bulked segregant analysis (BSA) was followed using microsatellite markers flanking the three putative candidate genes viz., an Acetolactate Synthase (ALS) on chromosome 6 and two Acetohydroxy Acid Synthase (AHAS) genes, one on chromosomes 2 and another on chromosome 4. RM 6844 on chromosome 2 located 0.16 Mbp upstream of AHAS (LOC_Os02g30630) was found to co-segregate with herbicide tolerance. Cloning and sequencing of AHAS (LOC_Os02g30630) from the wild type, N22 and the mutant HTM and their comparison with reference Nipponbare sequence revealed several Single Nucleotide Polymorphisms (SNPs) in the mutant, of which eight resulted in non-synonymous mutations. Three of the eight amino acid substitutions were identical to Nipponbare and hence were not considered as causal changes. Of the five putative candidate SNPs, four were novel (at positions 30, 50, 81 and 152) while the remaining one, S627D was a previously reported mutant, known to result in Imidazolinone tolerance in rice. Of the novel ones, G152E was found to alter the hydrophobicty and abolish an N myristoylation site in the HTM compared to the WT, from reference based modeling and motif prediction studies.

**Conclusions:**

A novel mutant tolerant to the herbicide “Imazethapyr” was developed and characterized for genetic, sequence and protein level variations. This is a HTM in rice without any IPR (Intellectual Property Rights) infringements and hence can be used in rice breeding as a novel genetic stock by the public funded organizations in the country and elsewhere.

**Electronic supplementary material:**

The online version of this article (doi:10.1186/s12284-017-0151-8) contains supplementary material, which is available to authorized users.

## Background

Weed management is labour intensive in irrigated rice, the most prevalent rice ecosystem. Owing to labour and water shortage, many South East Asian countries including Malaysia, Sri Lanka and Vietnam have shifted to direct seeded rice (DSR) from transplanted rice (Rao et al. 2007). In American and European countries, DSR accounts for 80–90% of total rice cultivated area (Hassan and Rao 1996). Due to predictions on increased frequency in the occurrence of water deficit/drought in Asia, DSR is expected to become popular in Bangladesh, Pakistan and India. In DSR, weed(s) are one of the major factors affecting rice production to an extent of 18–48% due to rice-weed competition for resources (Rao et al. 2007). This not only increases the cost of production due to increased labour cost for their management, but also affects productivity under situations of labour shortage. In India, the cost towards controlling the weeds accounts up to 30% of the total cost of cultivation (Rao et al. 2015). Moreover, the problem of weedy rice is being reported widely in DSR areas in India, for which herbicide tolerant rice varieties is one of the feasible and practical long term solutions (Rathore et al. 2013).

Herbicides primarily act by disrupting key enzymes/proteins involved in essential metabolic or physiological processes associated with growth and development of plants. Glyphosate, glufosinate, synthetic auxins, sulfonylurea, imidazolinones, triketones, isoxazoles, callistemone, cyclohexanediones, aryloxyphenoxypropionates and phenylpyrazolines are common herbicides for which herbicide tolerance (HT) mechanisms are well known and exploited for development of herbicide tolerant crops (Endo and Toki, 2013). Some of the herbicide tolerant crops have been developed by introducing mutations in the target site of herbicide action, whereas others have introduced genes detoxifying the herbicide molecule (Endo and Toki, 2013). Both the above mechanisms have been exploited in developing HT transgenic crops while the former approach has been achieved through mutagenesis (non-GM approach) as well (Green and Owen, 2010). Non-GM HT crops have the advantage of easier registration/release for commercial cultivation as well as wider public acceptance.

Of the various herbicides, imidazolinones are the most widely targeted ones for developing herbicide tolerant crops through non-GM approach. Imidazolinones act by disrupting Acetolactate Synthase (ALS), and/or Acetohydroxy Acid Synthase (AHAS), enzymes involved in branched chain amino acid (valine, leucine and isolecine) biosynthesis (Singh and Shaner, 1995). Nucleotide and amino acid sequences of AHAS and mutations causing tolerance against imidazolinones are well characterized in *Arabidopsis* (Sathasivan et al. 1990 and 1991). Mutations in AHAS gene is reported to confer resistance to five groups of herbicides, namely, imidazolinones (Shaner et al. 1984), sulfonylurea (Chaleff and Mauvais, 1984), pyrimidinyl thiobenzoates (Stidham, 1991), triazolopyrimidine and sulfonylaminocarbonyltriazolinones (Gerwick et al. 1990). Although ALS/AHAS mutants tolerant to imidazolinone compounds have been developed in rice, wheat, sunflower, canola and maize (Tan et al. 2005), they are all protected by patents (Croughan 1998, 2002; Livore 2003; Livore et al. 2011). The present investigation was therefore undertaken with the objective of identifying novel Imazethapyr resistant rice lines through Ethyl Methane Sulphonate (EMS) induced mutagenesis which can be used without restriction in public funded rice breeding. We report here the identification and detailed characterization of one such stable herbicide tolerant mutant in rice for the first time in India.

## Methods

### Mutagenesis

One kilogram seeds of a drought tolerant upland rice variety, Nagina 22 (N22) was treated with 0.8% EMS to raise M_1_ (Mohapatra et al. 2014). N22 was chosen for mutagenesis for two reasons: 1. it is an upland variety sensitive to Imazethapyr spray; 2. EMS mutants in N22 have been developed in a national level consortium on rice functional genomics owing to its positive attributes such as drought tolerance and heat tolerance, etc. (Mohapatra et al. 2014; Amitha Mithra et al. 2016). The produce of all the M_1_ plants was bulked to generate a large M_2_ population of about 100,000 M_2_ plants, which was used for screening for herbicide tolerance.

### Screening for Herbicide Tolerance

**Fig. 1:**
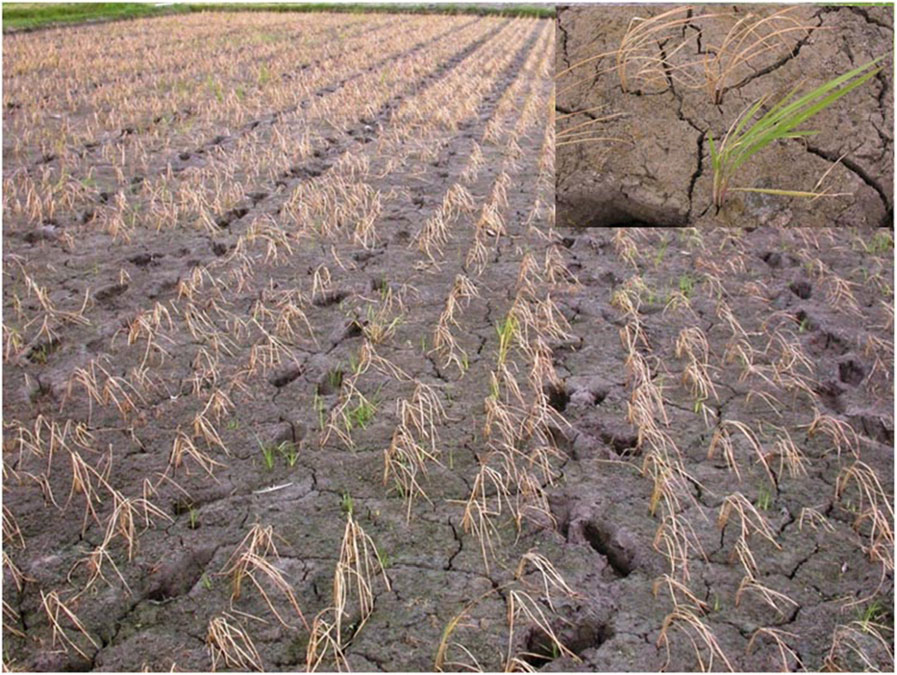
Overall view of imazethapyr treated field and identification of resistant plant (inset) in M_2_ generation

All the M_2_ seeds, giving rise to approximately 100,000 M_2_ plants were grown under field conditions by following recommended agronomic practices. Non-selective broad spectrum herbicide, Imazethapyr, available as Pursuit™ was sprayed @2.5 ml/lit on all plants on 30 days after sowing (DAS). Observation on survival of the mutants was made on 15th day after spraying. Putative resistant plants were identified based on retention of greenness of leaves and absence of drying symptoms. Particularly, green plants surrounded by dried plants were selected to ensure that the plants in the specific area had received adequate quantity of herbicide, thus minimizing the chances of escape (Fig. 1).

### Generation Advancement

**Fig. 2:**
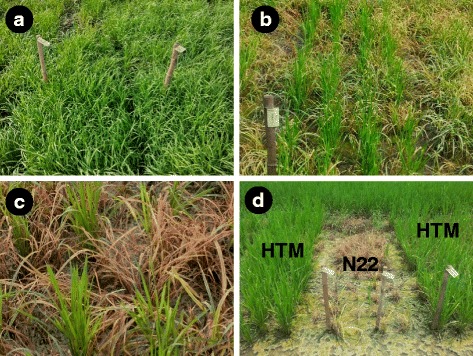
Mutant progenies 15 days after imazethapyr treatment in M_4_ generation. **a**: HTM-N22 (25th Day after sowing and at the time of treatment); **b**: HTM-N22 emerging from the weeds 15 days after treatment; **c**: Efficacy of the weed control (20th day after treatment); **d**: Nagina 22 killed along with the weeds

Putative resistant plants were rescued and replanted in pots, harvested separately and forwarded to M_3_ generation. M_3_ generation was raised under field conditions in progeny to row design along with the suitable controls viz., wild type (WT) N22 and IR 64 and, sprayed with Imazethapyr on 30th DAS for reconfirming the HT reaction. The HT plants were again harvested individually (single plants) and raised as progeny rows in M_4_ generation along with N22 and IR 64. Herbicide spray was applied as in the M_2_ and M_3_ generation (Fig. 2) and individual progenies were harvested in bulk. The progeny rows were validated again for herbicide tolerance in M_5_ generation under field conditions. One of the mutants, consistently breeding true over sucessive generations was finally selected and named “HTM-N22”. DUS (Distinctness, Uniformity and Stability) characterization of the mutant HTM-N22 (HTM) vis-à-vis the wild type N22 was carried out for 40 DUS traits to confirm the phenotypic similarity of the mutant to the wild type.

### Genetic Analysis of Herbicide Tolerance

To analyze the inheritance pattern, the herbicide tolerant mutant, HTM-N22 was crossed with Pusa 1656, a wild type parent for the herbicide tolerance trait. The F_2_ population comprising of 254 plants was raised in the field and screened for tolerance to Imazethapyr @ 2.5 ml/lit spray on 30th DAS. Data on number of plants dried and survived was recorded after 15 days of herbicide spray. The observed phenotypic data of the F_2_ generation was analyzed for the goodness of fit with the expected Mendelian segregation ratio using chi-square (*χ* ^2^) test (Fisher, 1936).

### Confirmation of Genomic Similarity Between the Mutant and its Wild Type and Molecular Mapping of Herbicide Tolerance

Since EMS mutagenesis is known to induce multiple mutations in the genome (Abe et al. 2012), testing with a large number of microsatellite markers is recommended before proceeding with mapping and or other applications (Wu et al. 2005; Lima et al. 2015). Hence, the herbicide tolerant mutant identified was subjected to comparative microsatellite analysis with the WT, N22 following the earlier protocol (Lima et al. 2015). A total of 127 genome wide SSR (Simple Sequence Repeat) loci including nine HvSSRs (hyper variable SSRs), which covered all 12 rice chromosomes, were used to study their allelic constitution between the WT and the HTM. The SSR fragments were amplified and separated on 3.5% agarose gels, and stained with ethidium bromide. The gels were documented using VersaDoc Imaging System Model3000 (Bio-Rad Laboratories, Inc. USA) and allelic pattern was scored based on the amplicon size.

In order to map the gene(s) responsible for herbicide tolerance in the HTM, Bulked Segregant Analysis (BSA) proposed by Michelmore et al. (1991) was carried out using SSR markers in the vicinity of ALS gene which is well established as the target of the herbicide, Imazethapyr. BLAST search against rice Pseudomolecule ver. 7.0, revealed that rice has three candidate genes namely, LOC_Os06g51280*,* encoding ALS, LOC_Os02g30630 and LOC_Os04g32010, both encoding AHAS. Parental polymorphism survey was conducted between HTM and Pusa 1656, using SSR markers located within 0.5 Mb region of the above three loci.

DNA (isolated from leaves sampled before herbicide spray) from 10 tolerant and 10 sensitive plants from the F_2_ generation, which was used for inheritance studies and phenotyped based on mortality upon herbicide spray, were pooled in equi-molar concentration to constitute the resistant and susceptible bulks, respectively. Based on inheritance pattern observed, the markers, which were polymorphic between the parents, and showing heterozygous (co-dominant) pattern in tolerant bulk and homozygous pattern in susceptible bulk, were considered to be putatively linked with the herbicide tolerance trait. The putatively linked markers were then used for genotyping of the individual F_2_ plants to study linkage relationship.

The genotypic and the phenotypic data of the F_2_ population were analyzed for segregation distortion, if any, using chi-square (*χ* ^2^) test for goodness of fit. Linkage between the putatively linked marker and the target gene was estimated using genotypic data and the phenotypic data with the help of MAPMAKER v3.0 software (Lander et al. 1987). Linkage was considered significant if the Logarithm of Odds (LOD) score was ≥3.0. The Kosambi mapping function (Kosambi, 1943) was used to convert recombination frequency into map distance between the marker and the target.

### Identification of Causal Gene and Validation in the Mapping Population

The candidate gene which was linked to the microsatellite marker and co-segregated with herbicide resistance was amplified using the flanking markers in both the mutant and the wild type. The amplified fragments were purified, cloned in pGEM-T vector, propagated in *E.coli* and were sequenced (Eurofins Genomics India Pvt. Ltd., India). Obtained nucleotide sequences were compared with reference *japonica* genome by multiple sequence alignment and all the nucleotide sequences were translated into amino acid sequences. Comparisons were made between the amino acid sequences of mutant plants and wild type, and then compared with the reference *japonica* genome sequence in the NCBI/TIGR database.

All the non-synonymous substitutions identified between the WT and HTM, and barring those with Nipponbare, were chosen for validation in the mapping population. Two sets of primers were designed to amplify the genomic regions that covered all the five single nucleotide polymorphisms of the candidate gene. The PCR product from each of the 96 genotypes (two parents as well as a subset of 94 individuals from F_2_ population) was purified and sequenced directly using Sanger’s dideoxy method on an automated capillary-based DNA sequencer (ABI 3730xl, Applied Biosystem, USA) in both forward and reverse directions as per standard ABI sequencing protocol. The trace files were base called and checked for quality using the internal software of the sequencer (ABI sequencer software v5.4). Trimming option was used to edit the poor quality sequences and Quality value (QV) of 20 was fixed for base calling for 99% accuracy. Contig formation using forward and reverse sequences, and sequence alignment so as to call SNPs were performed using BioEdit software.

### Protein Modelling of the Causal Gene Between the WT and the HTM

Since neither X-ray crystallographic nor NMR (Nuclear Magnetic Resonance) spectroscopy) structure of the protein encoded by the causal gene were available, reference based structural modeling of the protein from WT and HTM was carried out using Modeler 9.17 programme (Webb and Sali, 2014). The developed 3-D model structure was verified with veriry3D. The motifs in the WT and HTM protein sequences were predicted using PROSITE programme (http://prosite.expasy.org/prosite.html; date of access 11th Nov. 2016).

## Results

### Mutant Recovery

A mutant population of about 100,000 single M_2_ plants generated by bulking the progeny of M_1_ plants raised from about 1 Kg of EMS treated N22 seeds were grown in the field and sprayed with herbicide, Imazethapyr. Three mutant plants putatively resistant to Imazethapyr were identified from the field in the M_2_ generation. However, only a single mutant plant was found to be tolerant to subsequent herbicide spray in the re-potting experiments which was named as, 'HTM-N22'. Tolerance against the herbicide imazethapyr exhibited by the mutant HTM-N22 (HTM) was confirmed across subsequent filial generations up to M_5_ and found to be true breeding (Figs. 1 and 2).

### Genetic Analysis of Herbicide Tolerance

Phenotyping of F_2_ progenies for their tolerance to the herbicide (Imazethapyr) could classify them into two distinct groups namely, herbicide tolerant and susceptible plants, based on survival after herbicide spray. Of the 254 individual F_2_ plants, 197 were tolerant to Imazethapyr spray while 57 were sensitive. The frequency distribution of F_2_ population with respect to herbicide tolerance suggested a dominant monogenic inheritance pattern. The observed data showed a goodness of fit to Mendelian segregation ratio of 3:1 ratio (*p* = 0.3463), when tested by *χ* ^2^ test, indicating that the herbicide tolerance trait in HTM is controlled by a single dominant gene (Table 1).

**Table 1:** Segregation pattern of herbicide tolerance in F_2_ population from cross Pusa 1656/HTM-N22

Cross	Total no. of plants	No. of F_2_ plants		Expected Genetic ratio	*χ* ^2^ value	*P*-value
		Survived	Died			
Pusa 1656/HTM-N22	254	197	57	3:1	0.887	0.3463

### Genomic Integrity of the HTM in Comparison With the WT

Genotyping of the HTM and N22 with 127 SSR markers including 9 HvSSRs (Additional file 1: Table S1A and Additional file 2: Figure S1) revealed complete monomorphism, which confirmed the high degree of genetic similarity between the mutant and the WT. The mutant was also identical to the WT for all the 40 DUS characteristics as well as agro-morphological traits (Additional file 3: Table S1B), reiterating the results of marker analysis (Additional file 4: Table S1C and Additional file 5: Figure S2).

### Molecular Mapping of Causal Mutation

Out of the 20 SSR markers selected from the genomic regions flanking the three putative candidate genes (Table 2), three markers namely RM20767 for ALS (LOC_Os06g51280), RM 6844 and RM5749 for AHAS in chromosomes 2 and 4 (LOC_Os02g30630 and LOC_Os04g32010) were found to be polymorphic between the parents, Pusa 1656 and HTM.

**Table 2:** SSR markers identified in the vicinity of candidate genes for polymorphism survey between the HTM-N22 and Pusa 1656

Candidate gene	SSR	Position (bp)	Primers (Forward/Reverse)	Polymorphism
Aceto-lactate synthase (LOC_Os06g51280 at 31049803 in chromosome 6)	RM20765	30593302	CCAGCTCACCTCAGCTTCATCAGC CATCACCATCACCACCACCATGC	monomorphic
	RM20767	30593847	TCGATCGATCCTAGACTCCTTCC GACTCCACGAACAGCAGGTTAGC	polymorphic
	RM20768	30611463	CAGGGAATAAACAGGAGGAAGAGG CCAACCTCAACCTAGTTAACCTCACC	monomorphic
	RM20769	30653429	CAGATGCGGAGGATGAAGAGC CTTCCGGAATTTCAACTCAACG	monomorphic
	RM20771	30665825	CAAACCTGCGTCTCTGTCTCTCTCC GACGAGCACGACCCATCACC	monomorphic
	RM20773	30692412	TTGCCAATATTCCCTCCAGTGC GTTGTGTTGGGACCTTGATTCG	monomorphic
AcetoHydroxy Acid Synthase (LOC_Os02g30630 at 18236025 bp in chromosome 2)	RM 6844	18169413	AGTCCAAGAAAGGCACGAGAGG CTGCATCGAAGAAGAAGAAGAAGC	polymorphic
	RM13263	18187191	AAGATTGCACACTGGTGTTCTCC AGAAGAGCCGGTCTTTGTCTCC	monomorphic
	RM13264	18192480	ATCTCCATCGTCTCCTTCCTTGG CGTACAGCCATATCCAGCAAACG	monomorphic
	RM13267	18220791	TGAGGCGACGACCACCTTCG CCAAAGCCGCAGGTTAAGCATCC	monomorphic
	RM13268	18223511	CCCAAACATCCAATACGACACC GACGAGCCACCACGTTAGTACG	monomorphic
	RM13269	18237768	GCTTCGGTAATTTGGTTTCGTGATCC GACCACGTGACGTTCCAAGACG	monomorphic
AcetoHydroxy Acid Synthase (LOC_Os04g32010 at 19169130 bp in chromosome 4)	RM16844	19095807	CACCGACTGGTTCGTCTACAGG GAGAAGATATGCAGGTGGAACTGG	monomorphic
	RM16850	19172772	TGCTTGTAAGAGAGGTCAAGAGAGG CCCACCATCTCGTAGAGCTAACC	monomorphic
	RM16867	19452211	GGAACGTAGCTGAAGTCATGAACC ATGAGCCTTGCTTGCTGTAGTCC	monomorphic
	RM16873	19485808	GCATATGCATGCAGGAATTGACC TGCACTCCAGCATTAAAGAACAGG	monomorphic
	RM1205	19643755	CAATCACAGAGCAACACGTACCC GCAGAGGCAGCTGAGAAGTATAGC	monomorphic
	RM1359	20041155	CGACTTGCCAAAGGTCAACG GATTCTACGGGCCACAAGTCC	monomorphic
	RM3643	20128692	GCTAAGCTAATCTGACCGGATCTACG GATGGGCCGATTAACAAATTCC	monomorphic
	RM5749	20131193	GCTCGTTTCTCTCGATCACTCG GCAAGGTTGGATCAGTCATTTCG	polymorphic

**Fig. 3:**
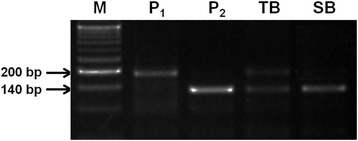
F_2_ population of HTM-N22-/Pusa 1656 tested by BSA with polymorphic SSR marker, RM 6844 in the vicinity of candidate gene, AHAS on Chromosome 2

**Fig. 4:**
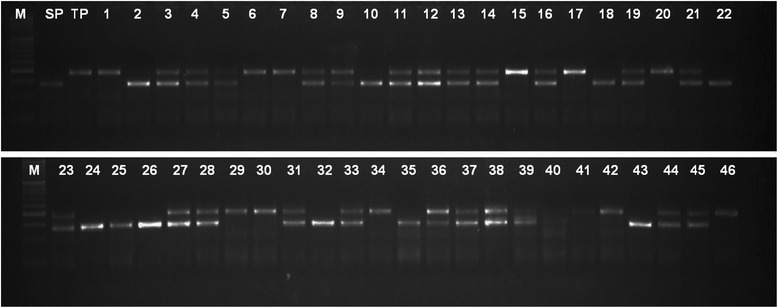
A representative gel picture showing the segregation pattern of RM 6844 in the F_2_ mapping population from the cross, Pusa 1656/HTM-N22. M -50 bp Ladder, SP – Herbicide susceptible parent, Pusa 1656; TP- Herbicide tolerant mutant, HTM-N22; 1– 46 progenies in F_2_ population

BSA was carried out using the three polymorphic markers between Pusa 1656 and HTM, with the herbicide tolerant and susceptible bulks, constituted from the plants of respective F_2_ populations, based on their survival on Imazethapyr spray. In the BSA, only one SSR marker, RM6844, was able to differentiate the herbicide tolerant and susceptible bulks and also exhibit the expected pattern, similar to the parents, while the remaining two markers failed to differentiate the herbicide tolerant and susceptible bulks. In case of RM6844, the herbicide tolerant bulk was heterozygous and the susceptible bulk was homozygous indicating that it is putatively linked with the herbicide tolerance trait (Fig. 3). The genotyping of 254 F_2_ individuals from the cross, Pusa 1656/HTM was undertaken with the putatively linked marker, RM6844 for molecular mapping of the gene governing herbicide tolerance (Fig. 4).

The goodness of fit for the genotypic segregation of the F_2_ population for the marker loci RM6844 was tested using *χ* ^2^ test against the expected ratio 1:2:1. The observed ratio fitted well with the expected ratio with respect to RM6844 marker with a p-value of 0.4392 (Table 3). Linkage analysis between phenotypic data on survival upon herbicide spray with genotypic data from RM6844 marker of 254 individuals in the F_2_ population from the cross, Pusa 1656/HTM revealed a genetic distance of 1.2 cM (LOD score - 51.89) between the causal gene and the marker.

**Table 3:** Genotypic segregation of F_2_ population from the cross, Pusa1656/HTM-N22 for RM6844 marker

Cross	Total no. of plants	Allele (observed)			Expected Genetic ratio	*χ* ^2^ value	*P*-value
		Mutant type	Heterozygote	WT			
Pusa 1656/HTM-N22	254	55	135	64	1:2:1 (63.5:127:63.5)	1.646	0.4392

### Sequence Analysis of the Causal Gene in the HTM and WT

**Fig. 5:**
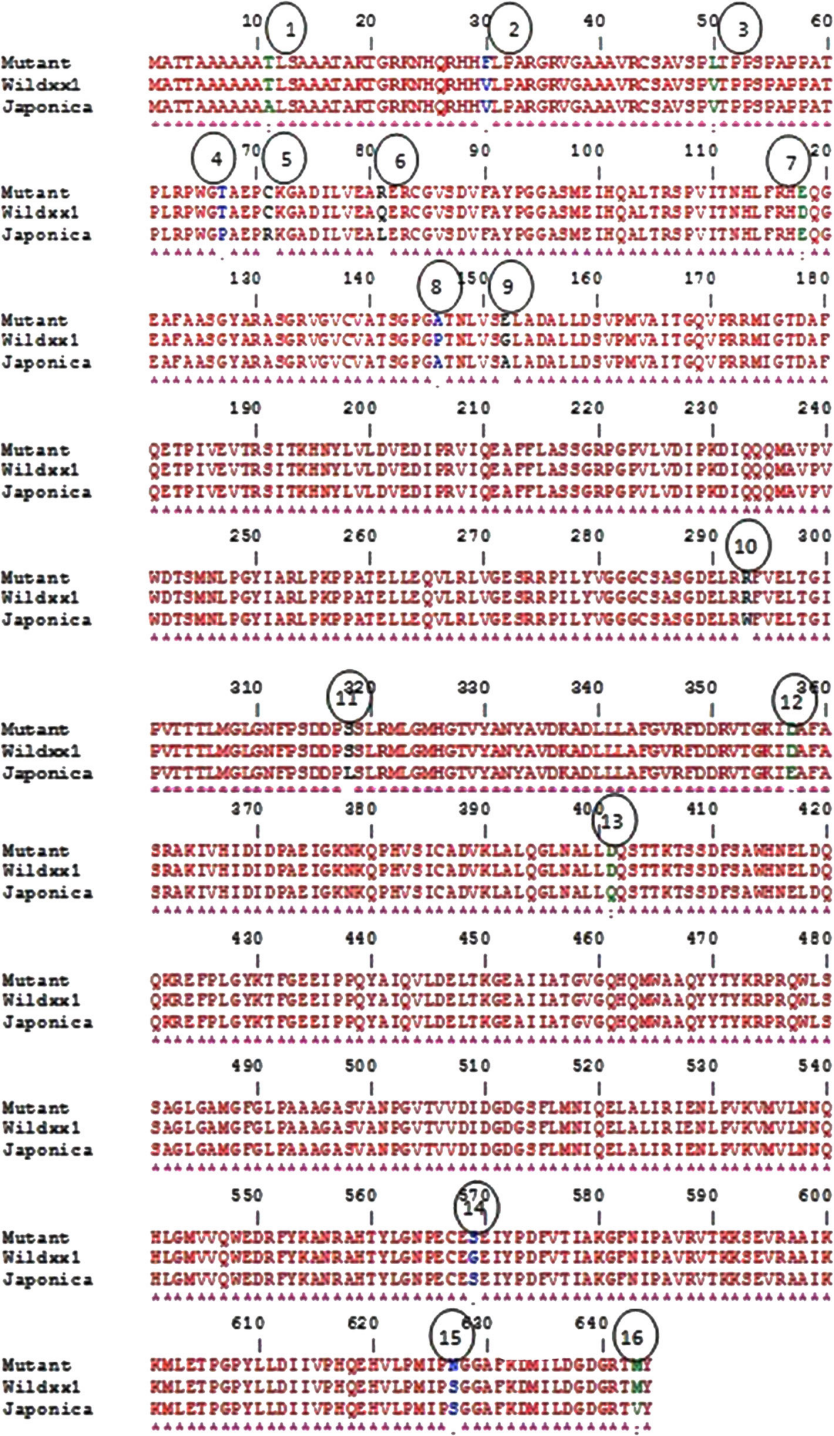
Amino acid sequence alignment of AcetoHydroxy Acid Synthase (LOC_Os02g30630) between wild type Nagina 22 and herbicide tolerant mutant along with Nipponbare

The locus, AHAS (LOC_Os02g30630) was amplified from both the HTM and the WT by designing appropriate primers (Forward: TCGCCCAAACCCAGAAA; Reverse: ACATCATAGGCATACCACTCTT), cloned and sequenced. Since LOC_Os02g30630 encoding AHAS is an intronless gene, it was amplified and sequenced in a single reaction (both forward and reverse) using a 50 cm long capillary. Nucleotide sequences of HTM and WT were translated to amino acid sequence and compared with each other as well as Nipponbare (*japonica* genome) as reference (Fig. 5). The sequence analysis revealed a total of 16 point mutations which resulted in amino acids substitutions across the three genotypes (Table 4). Out of these, three amino acids substitutions (at positions 30, 50 and 627) were specific to the HTM when compared to WT and Nipponbare, while eight changes (at positions 11, 67, 71, 293, 318, 357, 400 and 643), could be ascribed to the differences in subspecies – *japonica* vs. *aus* genotypes (WT and HTM). Interestingly, at three positions (118, 146 and 569) the amino acids in HTM were identical to the Nipponbare but different from the WT. At positions 81 and 152, the amino acid substitutions in HTM were completely different from references, WT as well as *japonica*. Thus the non-synonymous mutations in positions 30, 50 and 627 which were exclusively different between HTM and WT as well as those different from both WT and Nipponbare (81 and 152) were considered as potential causal mutations.

**Table 4:** Summary of amino acid substitutions in HTM-N22 as compared to WT with Nipponbare as reference

Amino acid position	Amino acid substitutions in			Remarks on amino acid substitutions in the HTM-N22 with respect WT and Nipponbare
	HTM-N22	WT	Nipponbare	
11	T	T	A	N22 type
30	F	V	V	Mutation
50	L	V	V	Mutation
67	T	T	P	N22 type
71	C	C	R	N22 type
81	R	Q	L	Different from both references
118	E	D	E	Nipponbare type
146	A	P	A	Nipponbare type
152	E	G	A	Different from both references
293	R	R	W	N22 type
318	S	S	L	N22 type
357	D	D	E	N22 type
400	D	D	Q	N22 type
569	S	G	S	Nipponbare type
627	N	S	S	Mutation
643	M	M	V	N22 type

### Validation of Potential Causal Mutations in the Mapping Population

Since SSR marker, RM6844 found to be linked to the causal gene, LOC_Os02g30630, encoding AHAS was 1.2 cM away, sequencing of the causal gene was done in a subset of mapping population (94 F_2_ individuals) for further validation. Two pairs of primers, one for amplifying region comprising amino acids 30, 50, 81 and 152 (Forward: CATCACCCACCATGGCTAC; Reverse: CTATGGGCGTCTCCTGGAA) and the other for the amino acid 627 (Forward: CAGTCCGTGTAACAAAGAAGAG; Reverse: GGGTCATTCAGGTCAAACATAG) were designed and PCR products were purified and sequenced. The sequencing results of all the five putative mutations were in complete agreement with the expected phenotyping data. Though, we could confirm the causal gene, the causal mutation could not be ascertained as we could not identify any recombinants in the material tested among the five potential causal mutations, owing to small population size.

### Reference Based AHAS Protein Modeling of WT and HTM

**Fig. 6:**
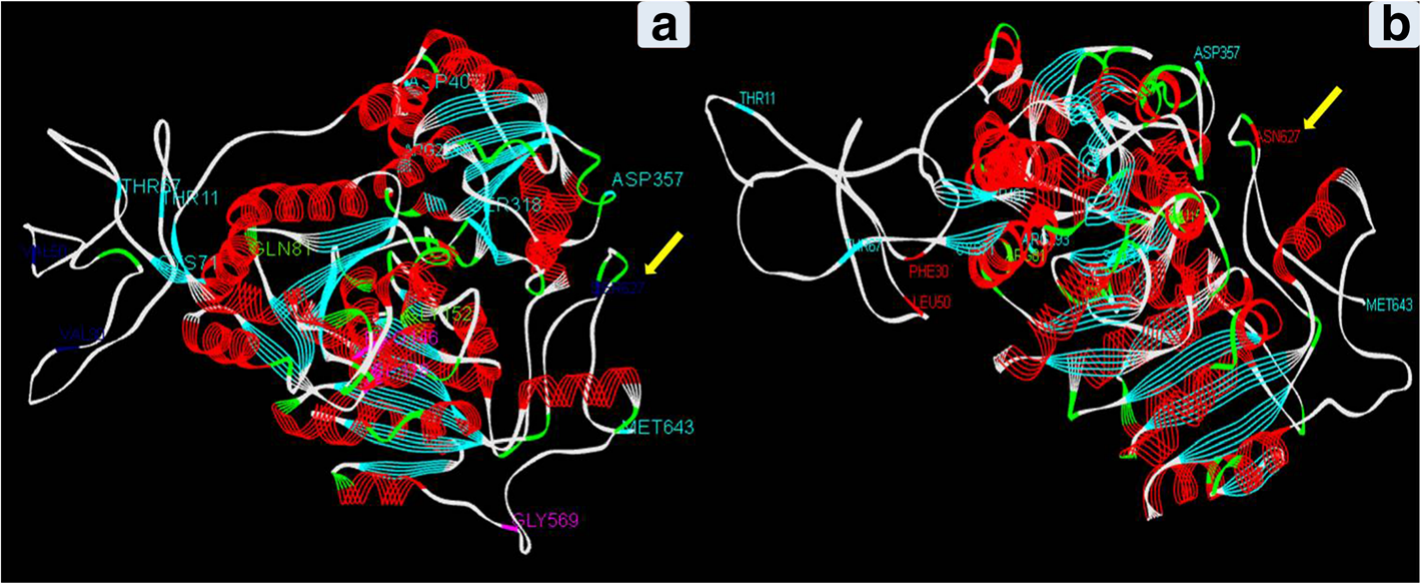
Line ribbon representation of the homology based protein model of WT (**a**) and HTM-N22 (**b**)

Three dimensional structure of AHAS protein in the WT was modeled based on four different templates, 3E9Y A (*Arabidopsis thaliana* ALS in complex with Monosulfuron), 1YBH A (*A. thaliana* ALS in complex with Sulfonylurea), 1JSC A (Yeast ALS as a target for herbicidal inhibitors), 1NOH A (Yeast ALS in complex with Sulfonylurea). BLASTP showed the corresponding identity with the *A. thaliana* templates to be 76%, while that of yeast to be 41%. The 3-D models of the AHAS from WT and HTM developed using Modeler had the 3D-1D score > = 0.2 score with the variation in WT and HTM in the range of 76.55 – 79.66% suggesting the models developed were appropriate. The DOPE (Discrete Optimized Protein Energy) score of the protein structure of WT and HTM was similar (-71451.57031 and -71297.08594 respectively) suggesting the high quality of the models. The superimposition of both the models did not reveal any major structural differences other than a coil generated in the HTM around position 627 which was not there in the WT (Fig. 6). Despite the major similarities observed between the WT and HTM, hydrophobicity chart showed a difference in hydrophobicity around 152nd amino acid (Additional file 6: Figure S3).

### Motif Prediction in WT and HTM AHAS Proteins

Functional motif prediction of AHAS protein in WT and HTM identified four distinct patterns viz*.,* protein kinase C phosphorylation, Amidation, N-myristoylation and Casein kinase II phosphorylation sites (Table 5). Comparison of WT and HTM for individual motifs predicted (within each of the four patterns) revealed abolition of a N-myristoylation site motif in HTM involving amino acids 152–157 which was present in the WT. Comparison of the amino acid sequences between WT and HTM (Fig. 5) revealed glutamic acid at 152nd position in the HTM while it was glycine in the WT. Thus in the HTM, a small non-polar aliphatic amino acid (glycine) was replaced by a polar (acidic) amino acid, glutamic acid abolishing an N-myristoylation site. In Nipponbare also the 152nd position was occupied by another small aliphatic and non-polar amino acid, alanine. Thus the motif prediction results were corroborated by the hydrophobicity chart (Additional file 6: Figure S3). Hence, the SNP/mutation in the 152nd amino acid could be the causal mutation conferring tolerance against imazethapyr in the HTM.

**Table 5:** Functional motifs predicted in ALS proteins of WT and HTM-N22

S. No.	Motif sites	WT		HTM-N22	
		Position of the amino acid	Features	Position of the amino acid	Features
1	PS00005 PKC_PHOSPHO_SITE Protein kinase C phosphorylation site	17 – 19	TaK	17 – 19	TaK
		20 – 22	TgR	20 – 22	TgR
		132 – 134	SgR	132 – 134	SgR
		218 – 220	SgR	218 – 220	SgR
		273 – 275	SrR	273 – 275	SrR
		319 – 321	SlR	319 – 321	SlR
		353 – 355	TgK	353 – 355	TgK
		404 – 406	TtK	404 – 406	TtK
		471 – 473	TyK	471 – 473	TyK
		590 – 592	TkK	590 – 592	TkK
2	PS00009 AMIDATION Amidation site	20 – 23	tGRK	20 – 23	tGRK
3	PS00008 MYRISTYL N-myristoylation site	136 – 141	GVcvAT	136 – 141	GVcvAT
		152 – 157	GLadAL		
		281 – 286	GGgcSA	281 – 286	GGgcSA
		282 – 287	GGcsAS	282 – 287	GGcsAS
		283 – 288	GCsaSG	283 – 288	GCsaSG
		299 – 304	GIpvTT	299 – 304	GIpvTT
		310 – 315	GNfpSD	310 – 315	GNfpSD
		324 – 329	GMhgTV	324 – 329	GMhgTV
		327 – 332	GTvyAN	327 – 332	GTvyAN
		490 – 495	GLpaAA	490 – 495	GLpaAA
		496 – 501	GAsvAN	496 – 501	GAsvAN
		563 – 568	GNpeCE	563 – 568	GNpeCE
4	PS00006 CK2_PHOSPHO_SITE Casein kinase II phosphorylation site	287 – 290	SgdE	287 – 290	SgdE
		407 – 410	TssD	407 – 410	TssD
		431 – 434	TfgE	431 – 434	TfgE
		449 – 452	TkgE	449 – 452	TkgE
		505 – 508	TvvD	505 – 508	TvvD

## Discussion

Among various methods of weed management in rice cultivation, chemical control using broad spectrum herbicides is an effective and economic alternative management strategy. A few herbicide tolerant genotypes have been developed using non-transgenic approach, primarily through induced mutagenesis, in crops viz*.,* maize, canola, wheat, rice and sunflower (Anderson and Georgeson, 1989; Swanson et al. 1989; Newhouse et al. 1992; Croughan 2002; Shaner et al. 1996; Al-Khatib et al. 1998) though transgenic approach is more prevalent (Green, 2009; Duke and Powles 2009). Imidazolinone herbicides, which include imazapyr, imazapic, imazethapyr, imazamox, imazamethabenz and imazaquin and belong to major class of Group B herbicides, are used commonly in soybean and pulse cultivation but not in rice owing to sensitivity of rice crop to this group of herbicides. In the present study, we have successfully identified an induced mutant in rice tolerant to a broad spectrum non-selective herbicide, imazethapyr, considering the ease in registering and commercializing the material developed through non-transgenic approaches.

The true breeding and monogenic nature of the herbicide tolerant mutant identified in the present study was evident from the results of progeny testing (Fig. 2), inheritance studies (Table 3), and SSR and DUS characterization (Additional file 2: Figure S1 and Additional file 1: Tables S1A and B). While genetic characterization of HTM using 127 markers, at an average distance of one marker per 3 Mb, revealed a very high degree of genetic similarity with the WT, Nagina 22, the causal gene sequencing results indicated multiple point mutations in HTM resulting in eight amino acid changes in a distance of just 1.88 kb (Fig. 5). EMS induced mutagenesis is known to induce multiple mutations and this is one of the reasons as to why DNA fingerprinting is recommended apriori to their genetic characterization (Wu et al. 2005; Lima et al. 2015). Since the causal gene for Imazethapyr tolerance has been identified in our study, it is possible to develop a CAPS marker using appropriate primers, (F: CATCACCCACCATGGCTAC; R: CTATGGGCGTCTCCTGGAA) and restriction enzyme, BstEII-HF. However, use of CAPS marker is tedious in marker assisted breeding (MAB). Hence, RM6844, a codominant SSR, located 0.16 Mb upstream of AHAS (LOC_Os02g30630) can serve as a robust marker for MAB of this trait.

AHAS in *Arabidopsis* comprises of 670 amino acids while in rice it is 644 amino acids long (Sathasivan et al. 1990; http://rice.plantbiology.msu.edu/). At least seventeen alleles of AHAS gene, harbouring mutations in amino acids at single or multiple positions such as, 121, 122, 124, 197, 199, 205, 206, 376, 574, 653 and 654 (with respect to *Arabisopsis*) and conferring tolerance to imidazolinone, have been reported across crops (rice, maize, canola, sunflower and wheat) and 87 weed species including prickly lettuce, pigweed, livid amaranth and weedy rice (Shaner, 1999; Duggleby and Pang, 2000; Heap, 2016 from http://www.weedscience.org/). The occurrence of multiple alleles and all of them conferring tolerance to Imidazolinone and/or sulfonylurea treatment suggests that mutations across AHAS could render them unresponsive to these groups of herbicides. In weeds, Ala122, Pro197, Ala205, Asp_376_, Arg_377_, Trp574, Ser653 and Gly_654_ are the eight confirmed target sites reported across genera and countries so far (http://www.weedscience.com/Mutations/MutationDisplayAll.aspx; accessed on 21 Nov 2016). In weedy rice alone, three mutations, G654E, S653D and A122T are known (Roso et al. 2010). Among them, the change S653D (S627D in rice) is common across crops and weeds (Croughan 2002) whereas the rice Clearfield events released in USA had mutation G654E (G628E in rice) as known from the patent search (US 20070028318 A1). Thus, the HTM rice, identified in the current study with multiple amino acid substitutions (Table 4), all different from the rice Clearfield event, and is free from IPR issues.

As compared to the previous results, the causal mutations in the HTM identified in our study are novel with the presence of five possible candidates in 30, 50, 81, 152 and 627 amino acid positions. The non-synonymous substitution, V30F is part of the chloroplast transit peptide (US patent 57311810) and hence could not be the causal mutation for Imazethapyr tolerance. Using I-TASSER based protein modeling, an additional coil was found to be present in V50L in the HTM (data not shown) which however could not be validated by the rigourous reference based modeling. Similarly, our *in-silico* analysis could not find any difference in protein structure due to Q81R. Thus, there are two potential causal mutataions in HTM namely, S627D which has been established beyond doubt (Croughan 2002; Heap, 2016) and G152E as indicated by protein modeling and motif prediction analyses (Table 5 and Additional file 5: Figure S2). The N-myristoylation site abolished in the HTM owing to G152E substitution is known to be involved in signal transduction under the environmental stress condition in plants (Podell and Gribskov, 2004). This latter mutation is so far not reported in any of the crop plants and weeds (Heap 2016), thus making HTM-N22, a novel resource for herbicide tolerance. However, in order to conclusively demonstrate the role of this point mutation leading to substitution G152E, further detailed experimentation is needed either through transformation of the ALS gene with this point mutation/targeted mutation through CRISPR/CAS9 or through isolation of intra-genic recombinants with only one mutation namely S627D and G152E, by generating a sufficiently large F_2_ population from the cross N22/HTM.

N- myristoylation is known to cause lipid modification of proteins resulting in their proper intracellular trafficking and correct sub cellular targeting (Bhatnagar et al. 2001). N-myristoylation facilitates protein folding and improves their thermostability (de-Jonge HR et al. 2000). N-myristoylation was also found to be important for proper functioning of *Arabidopsis* gene, *SOS3* (*Salt overly sensitive 3*) having important role in plant salt tolerance (Ishitani et al. 2000). About 29 predicted N-myristoylation sites were reported in SbASR1 gene of *Salicornia brachiata* (halophyte), which is known for enhanced salinity and drought endurance, as against two in rice (Tiwari et al. 2015).

The novel imazethapyr tolerant mutant which has been developed indigenously has opened up the possibilities of its extensive usage, without fear of infringement of any IPR, in the public rice breeding programmes in India and elsewhere. A stable herbicide tolerant source generated and characterized for the causal mutation and the gene linked marker developed for marker assisted selection in this study is expected to benefit the rice breeding programmes widely to protect rice cultivation from weeds and weedy rice. Already the breeding populations have been synthesized between HTM and CO51, Pusa Basmati 1121, Pusa Basmati 1509 and Pusa 44 and the progenies developed through the linked marker RM6844 are in the second or third generation of backcross. Polymorphism studies conducted between the HTM-N22 and several *indica* upland cultivars under cultivation viz., Sahbhagi dhan, Naveen, Pooja and SwarnaSub1 using RM6844 (Additional file 7: Figure S4) indicate the robust nature of the marker across diverse genetic backgrounds. The scope of this trait would be enormous if the upland rice system which is highly vulnerable to weed and weedy rice infestation, derives the benefit out of this gene and the identified marker.

## Conclusions

In the present study, a novel herbicide (Imazethapyr) tolerant mutant resource was developed by EMS mutagenesis approach from a drought tolerant variety Nagina22. The genetic analysis established herbicide tolerance in the HTM as a monogenic trait. The tightly linked marker identified in our study has proven to be helpful in introgression of this trait in popular rice cultivars. Since this is a novel HTM in rice without any IPR infringements, it can be used in rice breeding by the public funded organizations in the country and elsewhere.

## Additional Files

Additional file 1: Table S1A. SSR primers used for testing the genomic similarity between the WT and HTM-N22. (XLS 43 kb)

Additional file 2: Figure S1. Genotyping results of WT and HTM-N22 with selected SSR markers for testing genomic similarity. (PPTX 293 kb)

Additional file 3: Table S1B. DUS characterization of WT and HTM-N22 for testing genetic similarity. (DOCX 16 kb)

Additional file 4: Table S1C_DUS characterization for genetic integrity testing. (XLS 26 kb)

Additional file 5: Figure S2. Comparison of WT and HTM – N22 (A) Single Plant; (B) Panicle. (PPTX 336 kb)

Additional file 6: Figure S3. Hydrophobicity chart of WT (upper panel) and HTM-N22 (lower panel). (DOCX 586 kb)

Additional file 7: Figure S4. Polymorphism between HTM-N22 and upland cultivars for the herbicide tolerance trait linked marker, RM 6844. (PPTX 230 kb)
