# Supplementary material for: Development and Genetic Characterization of A Novel Herbicide (Imazethapyr) Tolerant Mutant in Rice (Oryza sativa L.)
Source: Rice (N Y). 2017 Apr 4;10:10. doi: 10.1186/s12284-017-0151-8 (PMC5380566; doi:10.1186/s12284-017-0151-8)
Supplement: Supplementary file 2 — Genotyping results of WT and HTM-N22 with selected SSR markers for testing genomic similarity. (PPTX 293 kb) [file 12284_2017_151_MOESM2_ESM.pptx]

## Slide 1
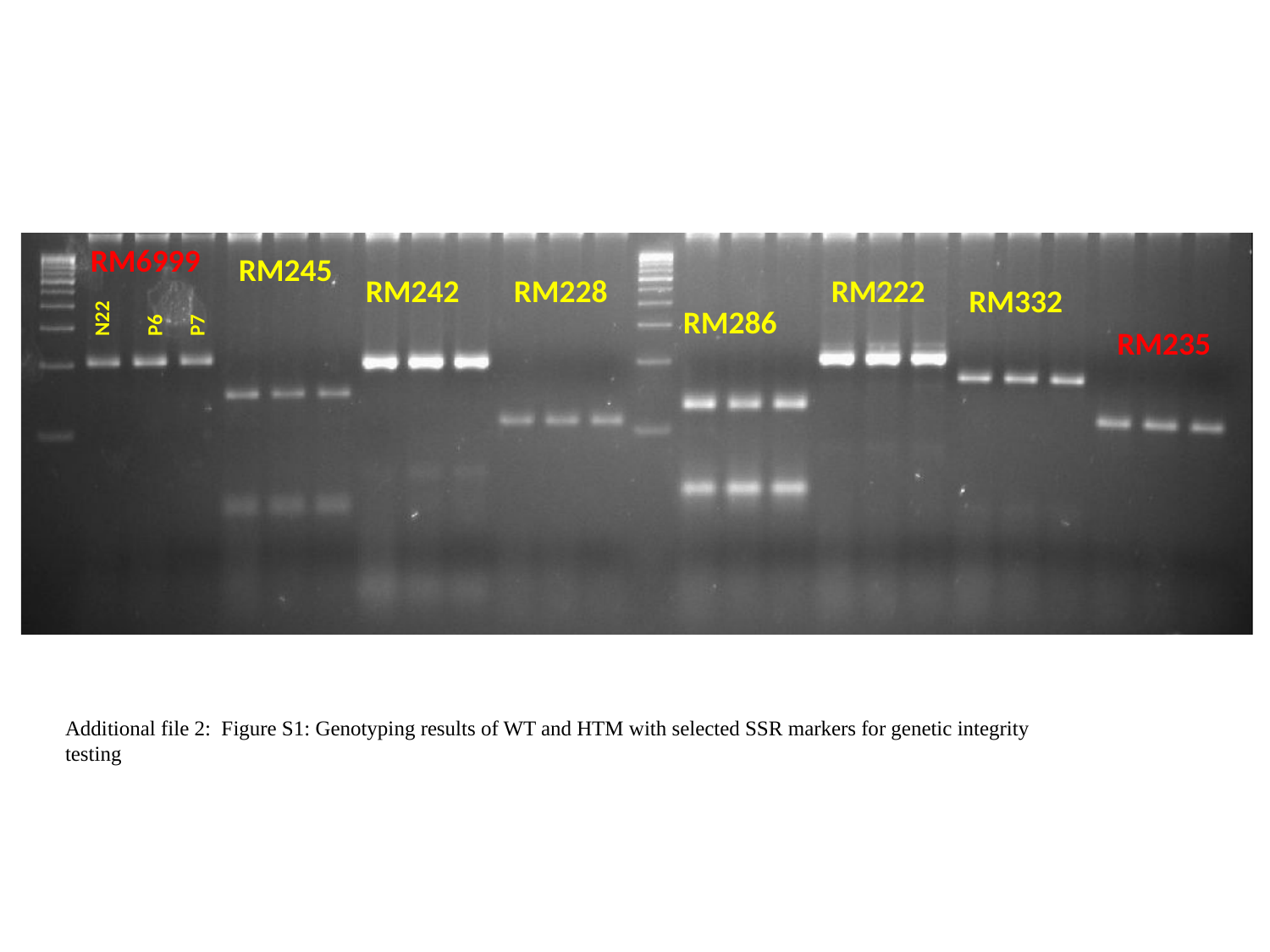

N22
P6
P7
RM6999
RM245
RM242
RM228
RM222
RM332
RM286
RM235
Additional file 2: Figure S1: Genotyping results of WT and HTM with selected SSR markers for genetic integrity testing
