# Supplementary material for: Development and Genetic Characterization of A Novel Herbicide (Imazethapyr) Tolerant Mutant in Rice (Oryza sativa L.)
Source: Rice (N Y). 2017 Apr 4;10:10. doi: 10.1186/s12284-017-0151-8 (PMC5380566; doi:10.1186/s12284-017-0151-8)
Supplement: Supplementary file 3 — DUS characterization of WT and HTM-N22 for testing genetic similarity. (DOCX 16 kb) [file 12284_2017_151_MOESM3_ESM.docx]

**Additional file 3: Table S1B.** Agro-morphological characters of N22 (Wild Type) as compared to HTM-N22

| **Traits** | **N22** | **HTM-N22** |
| --- | --- | --- |
| Days to 50% Flowering | 81.33 + 1.53 | 80.33 + 1.53 |
| Plant Height (cm) | 144.0 + 3.08 | 139.33 + 3.06 |
| No. of Tillers Per Plant | 16.5 + 2.38 | 16.8 + 3.83 |
| Panicle Length (cm) | 21.8 + 0.63 | 21.7 + 1.48 |
| Yield Per Plant (g) | 20.13 + 1.84 | 20.38 + 1.89 |
| Filled Grains Per Panicle | 126.60 + 16.38 | 129.00 + 10.82 |
| Spikelet Fertility % | 93.87 + 3.88 | 95.09 + 3.39 |
| 1000 Grain Weight (g) | 20.11 + 0.16 | 20.33 + 0.12 |
| Rough Rice Length (mm) | 7.74 + 0.28 | 7.71 + 0.28 |
| Rough Rice Width (mm) | 2.83 + 0.18 | 2.83 + 0.28 |

* All data are indicated as mean + SD
