# Supplementary material for: Development and Genetic Characterization of A Novel Herbicide (Imazethapyr) Tolerant Mutant in Rice (Oryza sativa L.)
Source: Rice (N Y). 2017 Apr 4;10:10. doi: 10.1186/s12284-017-0151-8 (PMC5380566; doi:10.1186/s12284-017-0151-8)
Supplement: Supplementary file 5 — Comparison of WT and HTM – N22 (A) Single Plant; (B) Panicle. (PPTX 336 kb) [file 12284_2017_151_MOESM5_ESM.pptx]

## Slide 1
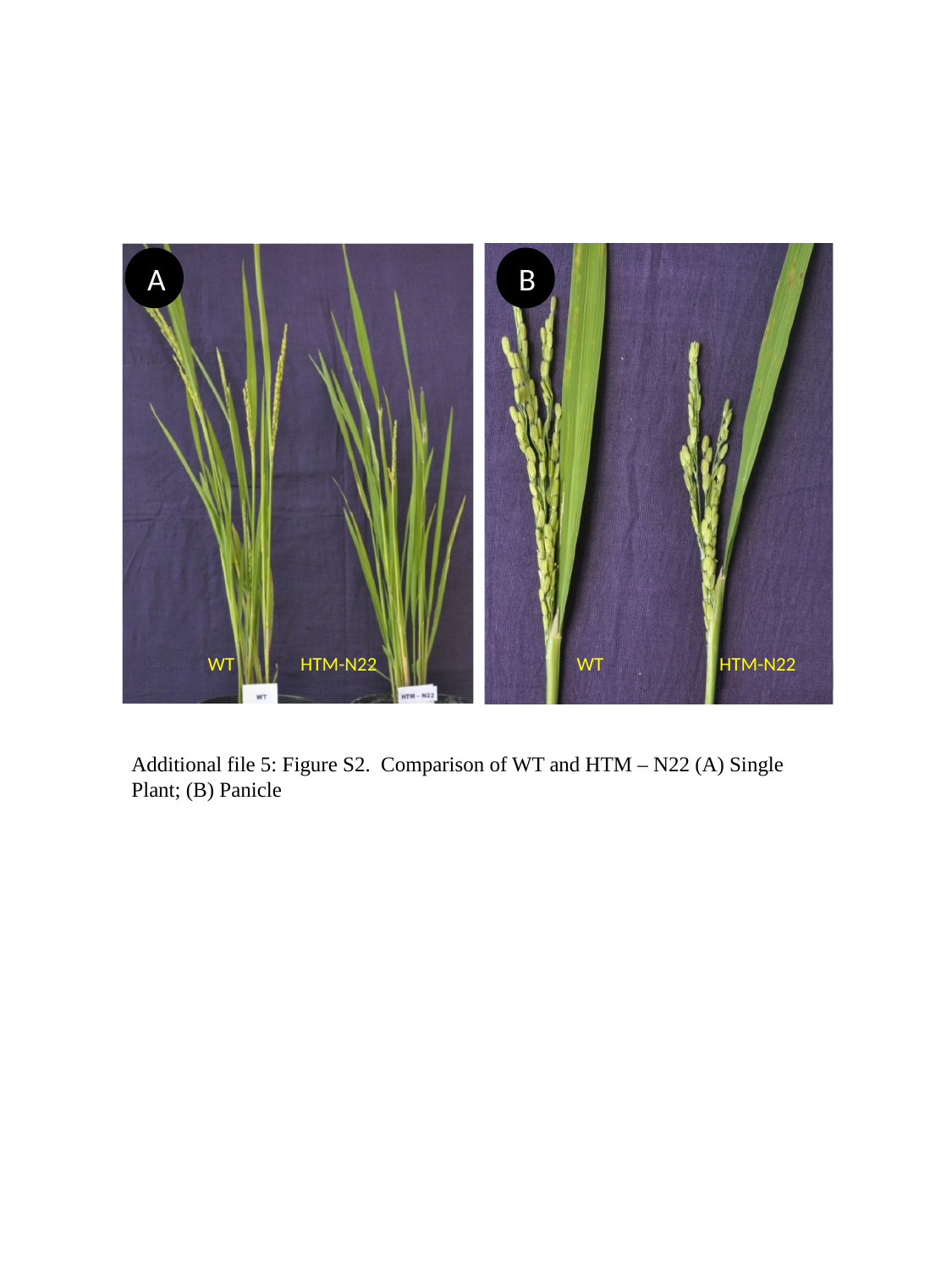

A
B
WT
HTM-N22
WT
HTM-N22
Additional file 5: Figure S2. Comparison of WT and HTM – N22 (A) Single Plant; (B) Panicle
