# Supplementary material for: Development and Genetic Characterization of A Novel Herbicide (Imazethapyr) Tolerant Mutant in Rice (Oryza sativa L.)
Source: Rice (N Y). 2017 Apr 4;10:10. doi: 10.1186/s12284-017-0151-8 (PMC5380566; doi:10.1186/s12284-017-0151-8)
Supplement: Supplementary file 6 — Hydrophobicity chart of WT (upper panel) and HTM-N22 (lower panel). (DOCX 586 kb) [file 12284_2017_151_MOESM6_ESM.docx]

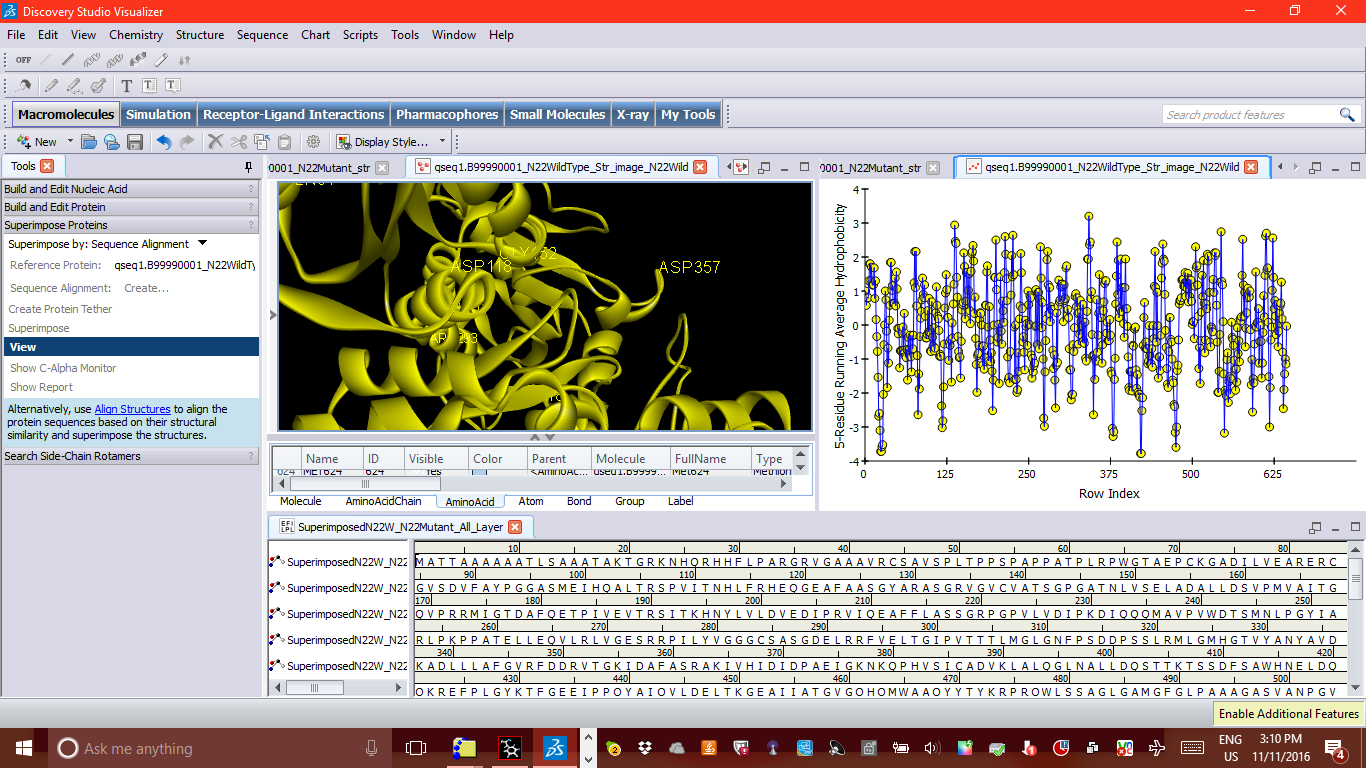


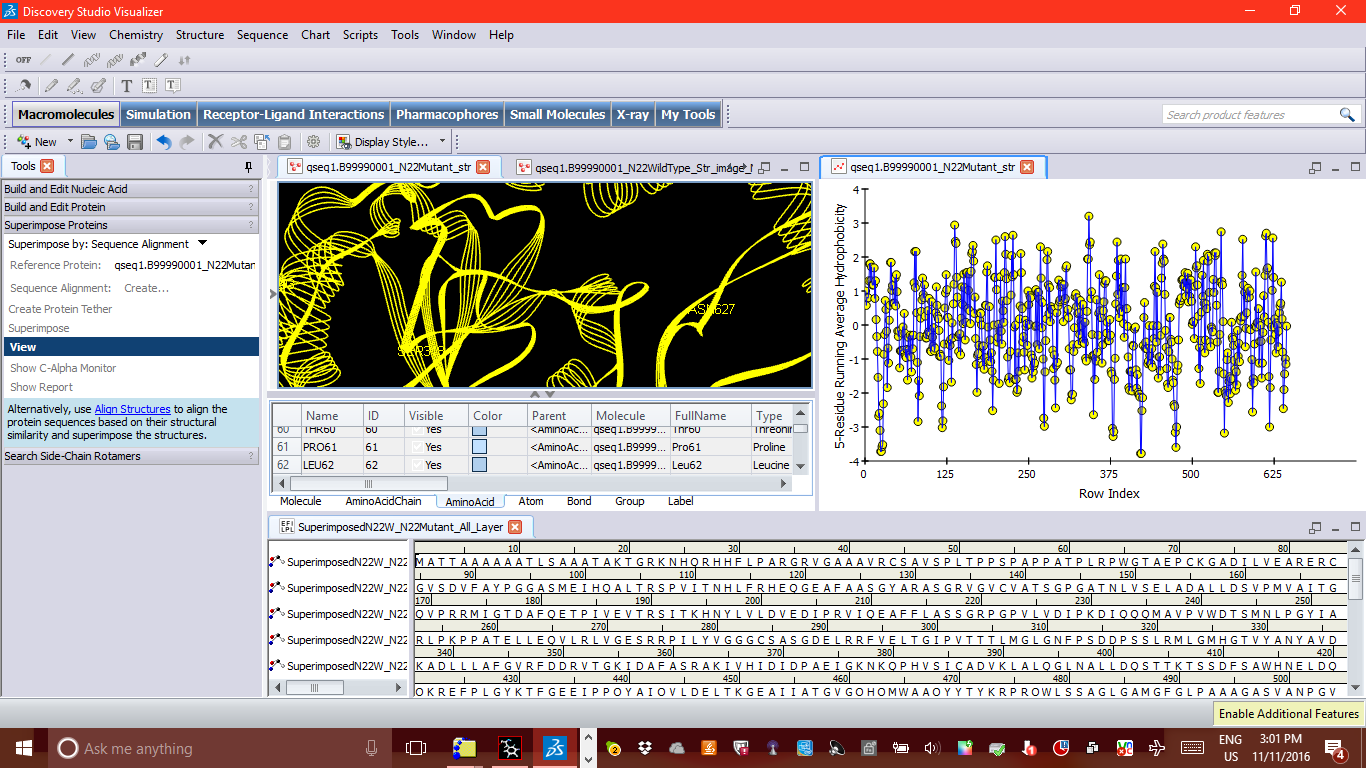


Additional file 6: Figure S3: Hydrophobicity chart of WT (upper panel) and HTM (lower panel). The differences in hydrophobicity are denoted by red circle.
