# Supplementary material for: Development and Genetic Characterization of A Novel Herbicide (Imazethapyr) Tolerant Mutant in Rice (Oryza sativa L.)
Source: Rice (N Y). 2017 Apr 4;10:10. doi: 10.1186/s12284-017-0151-8 (PMC5380566; doi:10.1186/s12284-017-0151-8)
Supplement: Supplementary file 7 — Polymorphism between HTM-N22 and upland cultivars for the herbicide tolerance trait linked marker, RM 6844. (PPTX 230 kb) [file 12284_2017_151_MOESM7_ESM.pptx]

## Slide 1
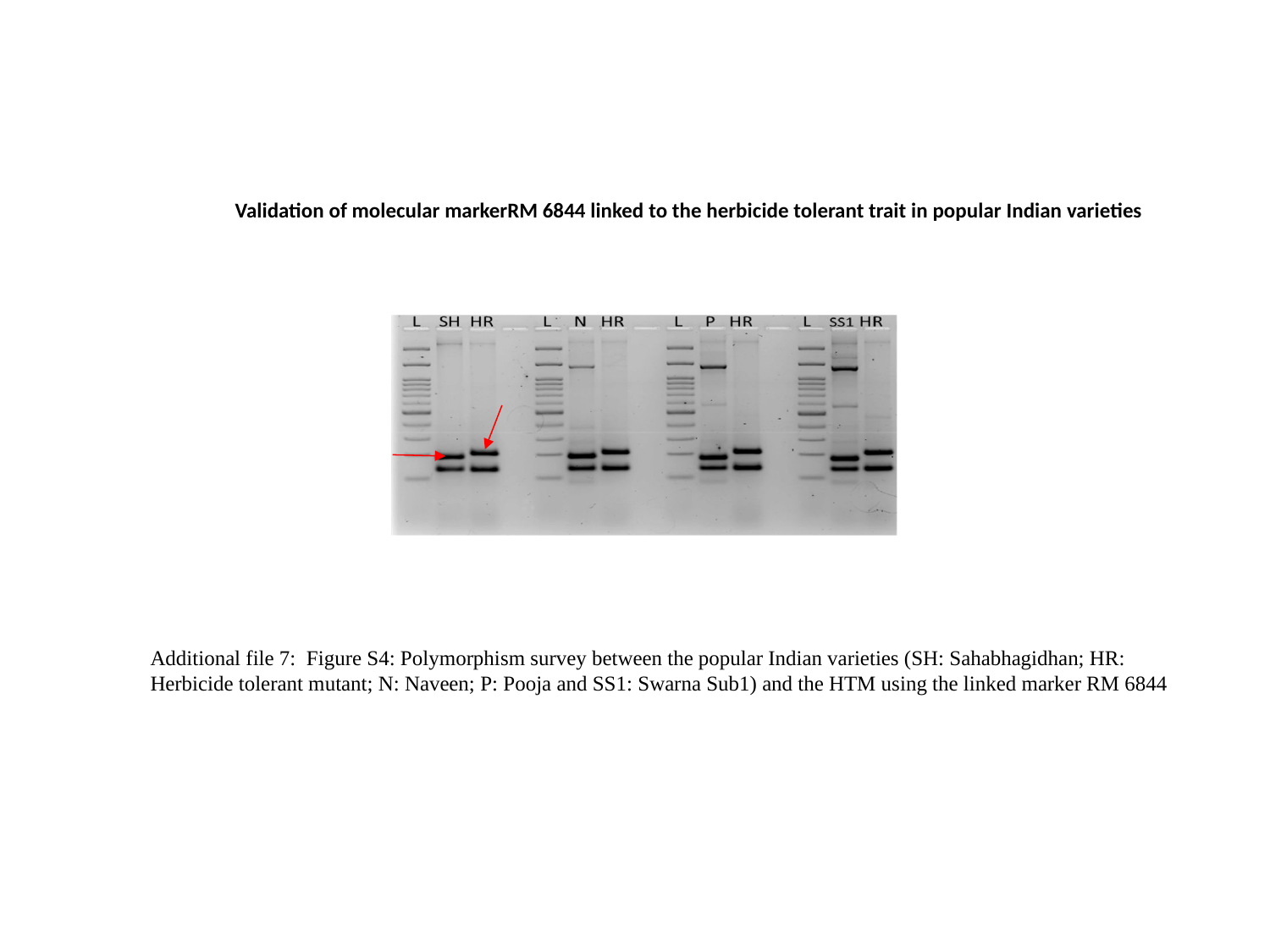

Validation of molecular markerRM 6844 linked to the herbicide tolerant trait in popular Indian varieties
Additional file 7: Figure S4: Polymorphism survey between the popular Indian varieties (SH: Sahabhagidhan; HR: Herbicide tolerant mutant; N: Naveen; P: Pooja and SS1: Swarna Sub1) and the HTM using the linked marker RM 6844
